# saltPAD: A New Analytical Tool for Monitoring Salt Iodization in Low Resource Settings

**DOI:** 10.5772/62919

**Published:** 2016-01-01

**Authors:** Nicholas M. Myers, Emmerentia Elza Strydom, James Sweet, Christopher Sweet, Rebecca Spohrer, Muhammad Ali Dhansay, Marya Lieberman

**Affiliations:** 1 University of Notre Dame, Department of Chemistry and Biochemistry, Notre Dame, USA; 2 Burden of Disease Research Unit, South African Medical Research Council, South Africa; 3 University of Notre Dame Department of Computer Science, USA; 4 University of Notre Dame Center for Research Computing, USA; 5 Global Alliance for Improved Nutrition (GAIN), Switzerland; 6 Division of Human Nutrition and Department of Paediatrics and Child Health, Faculty of Medicine and Health Sciences, University of Stellenbosch, South Africa

**Keywords:** Salt, Iodization, Adequately Iodized, Inadequately Iodized, Analytical Chemistry, Paper Analytical Device, Colorimetry, Wax Printing, Iodate, Iodometric Titration, Africa, Developing World, Low Resource Setting, Mobile Phone Application, Validation

## Abstract

We created a paper test card that measures a common iodizing agent, iodate, in salt. To test the analytical metrics, usability, and robustness of the paper test card when it is used in low resource settings, the South African Medical Research Council and GroundWork performed independent validation studies of the device. The accuracy and precision metrics from both studies were comparable. In the SAMRC study, more than 90% of the test results (n=1704) were correctly classified as corresponding to adequately or inadequately iodized salt. The cards are suitable for market and household surveys to determine whether salt is adequately iodized. Further development of the cards will improve their utility for monitoring salt iodization during production.

## 1. Introduction

### 1.1 The global health impact of iodized salt

Any analytical technology must be considered as part of a whole system: equipment purchase, calibration and maintenance; user training and skill verification; and purchase, storage and testing of consumable supplies. *In* developed world settings, these systems are supported by a network of technological and business infrastructure that ensures that laboratories can order instruments and supplies on credit, that a part can be mailed overnight to a trained repair person and that the most accurate and precise procedures for analyses are followed and documented by the operators. In developing world settings, these systems are often hindered by resource limitations, lack of trained staff to operate or fix equipment, rapid staff turnover, laboratories that lack power, refrigeration or bench space and slow and unreliable supply chains. How can new technologies developed in well-equipped laboratories in the developed world be tested for implementation in such different settings?

We first explore the background of salt iodization programmes and the analytical needs involved in monitoring such programmes in the developing world. The next section describes the main methods for monitoring iodine levels in iodized salt and their strengths and weaknesses for applications in low-resource settings; we then focus our discussion on a recently developed paper test card, using it as a case study for the early stages of transitioning a technology from laboratory to field implementation.

Iodine deficiency hinders cognitive development and growth of children and is the single greatest cause of preventable mental impairment. Salt iodization is accepted as the most effective and cost-effective strategy to combat global iodine deficiency. In 1994, the Joint UNICEF/WHO Committee on Health Policy recommended that all food-grade salt, used in household and food processing, should be fortified with iodine as a safe and effective strategy for the prevention and control of iodine deficiency disorders (IDD).[1] Thanks to global efforts by national governments, salt industries, international and national non-governmental organizations and scientists over the last two decades, iodine deficiency is no longer a common public health issue in many parts of the world. Currently, 76% of the global population has access to adequately iodized salt (defined as iodine levels ≥15 parts per million),[2] with the goal of reaching 90%. The number of iodine-deficient countries has fallen from more than 100 to just 25 today. [3] There must be a continued effort to reach the populations that are still negatively affected and to maintain the progress that has been made.

The main indicator of programme success has been coverage of iodized salt in households, generally derived from national household surveys using rapid test kits (RTKs). RTKs are simple, inexpensive, require no technical skills and provide immediate results to the user. However, they are non-quantitative, only detecting the presence of iodine and relying on the user's visual estimate of the concentration using a colour scale. UNICEF's Multiple Indicator Cluster Surveys (MICS) use RTKs to determine the presence of iodine in salt and the proportion of adequately iodized salt coverage, which limits the ability to assess the quality of salt iodization in many countries that rely on this survey alone. Several countries have started to back up the RTK method with the “gold standard” method of titration on a representative subset of samples in iodine or micronutrient-specific surveys, but this requires a dedicated laboratory, technical know-how, funding, transportation of the samples and time.

## 2. Methods to regulate salt iodization

In addition to determining whether iodized salt is available at the household level, it is critical to monitor the quality of iodized salt at various points along the supply chain. [4] This includes internal process controls at the fortification facility, external regulatory monitoring of fortification facilities to provide a check on quality, analysis at borders to ensure the quality of imported products, and market-based monitoring. [5] National regulations call for 20–100 micrograms of iodine per gram of salt,[6] which complies with WHO recommendations for populations to consume between 5–10 grams of salt per day. In 2014, the WHO amended its recommendation to reduce salt consumption to <5 grams per day,[7] which means that concentrations may need to be adjusted in the future, in light of national data regarding salt intake. The bodies responsible for regulation vary by country and sometimes by level of monitoring. For example, at factory level, regulation could be implemented by food and drug regulatory authorities, bureaus of standards, or dedicated bodies within Ministries of Trade. In some contexts, market-level monitoring may be conducted by the Ministry of Health, or sometimes at the provincial or district level. It is also necessary to test imported fortified salt, which can involve customs officials. Regulators take samples and test the salt using quantitative measures to ensure that it is within the acceptable range. Sampling in markets is necessary to prevent the fraudulent practice of packaging non-iodized salt with iodized salt labels, as a check on production quality control and because the iodine content of properly iodized salt is known to degrade over time.

## 3. Cost of Failure

Iodine deficiency disorders are caused by a nutritional deficit and, even if the condition is completely remedied by nutritional interventions, the underlying deficit cannot be forgotten. Normal diets for many populations do not provide enough iodine; therefore, fortification of commonly consumed foods, such as salt, oil, or sugar, is necessary to increase the iodine intake. Once a delivery platform is chosen, continual regulatory support is needed to help the correct product reach the population. Relaxation of legislative oversight and monitoring can cause serious harm in a short time. In 2006, Vietnamese lawmakers rewrote regulations so that salt for human consumption was no longer required to be iodized. As a result, large quantities of inexpensive non-iodized salt entered the market; the population's urinary iodine level fell from a healthy status in 2006 to a deficient one in 2009. [8] Provision of high-quality fortified salt must be treated as a long-term nutritional intervention and endowed with sustainable oversight and regulation.

## 4. Technologies for Iodine Analysis of Salt in Low-Resource Settings

### 4.1 Chemistry of common iodizing agents

The most common iodizing agents for food-grade salt are potassium iodate (KIO_3_) and potassium iodide (KI). Here, we focus on chemical analysis of salt that is fortified with iodate (IO_3_^−^). All countries in Africa, as well as most developing countries in tropical climates, use potassium iodate as an iodization source. [6] Potassium iodate is more expensive than potassium iodide, but also more stable under tropical conditions. It is therefore the major target for iodine analysis in low resource settings. All of the common chemical analysis methods for this chemical in iodized salt take advantage of the oxidizing power of iodate (E°_red_ = +1.20V) to generate coloured species. The various field analysis methods can be differentiated by the way in which the generation of a coloured species is tied to a determination of the quantity of iodate present in a salt sample.

### 4.2 Rapid test kits (RTKs)

Rapid test kits consist of dropper bottles of reagents, which are applied directly to portions of salt. The reagents contain iodide, acid and starch, to produce a visible blue colour when the salt sample contains iodate. Some kits provide a “recheck” solution, which can be used if the salt samples are unusually basic, and others provide a separate test solution for salt iodized with iodide rather than iodate. In each case, the user compares the intensity of the colour produced by the reagent solution to a printed colour scale or photographs of standard samples. These kits are simple, inexpensive and easy to operate, but they do not have the ability to quantify iodine in iodized salt. A study conducted by the WHO compared the ability of a commonly used RTK to distinguish adequately iodized salt (≥15 ppm I) from inadequately iodized salt and found only a 40% specificity for multiple observers. [9] When determining just the presence of iodine on salt, the test kit only had a 14% specificity for multiple users. These very high false-positive rates mean that many samples that are not adequately iodized are categorized as adequately iodized, so the RTK could not garner the WHO's recommendation as a viable technology. In a study undertaken by the South African Medical Research Council (Cape Town, South Africa) in collaboration with the WHO (Geneva), UNICEF (New York, USA), CDC (Atlanta, USA) and the University of Saint Pierre, Brussels, 10 existing RTKs were evaluated, showing progressive colour intensities with increasing iodine concentrations; however, these colour reactions were crude approximations of the iodine concentrations compared with the titration method. [10] Despite these studies, RTKs are still used to check household salt and by some manufacturers in low-resource areas.

### 4.3 Spectrophotometry

A number of handheld, transportable spectrophotometric devices exist to analyse iodized salt. Current validated devices that are commercially available rely on detecting the characteristic blue colour of the triiodide/starch complex[11]: iodide reduces the iodate from the salt sample in acidic conditions. The intensity of the blue colour is directly proportional to the iodine content of the salt sample. However, the triiodide/starch complex is unstable, so measurements must be made within a few minutes. The reagents needed for the colour generation must be packaged carefully as the iodide is subject to air oxidation and the starch can be broken down by bacteria or by exposure to acidic pH. The validated iCheck Iodine test kit (iCheck), which was recently introduced onto the market, consists of a portable spectrometer and disposable septum-sealed reaction and measuring vials containing starch solution, iodide and acid. [12] The consumables of the kits must be purchased from the manufacturer. The analyst uses a syringe to inject a portion of an appropriately diluted salt solution directly into a vial and withdraws and injects the other reagents to initiate the reaction. The blue colour is then read in a spectrometer after five minutes of reaction time. Other spectrometric devices, such as the WYD Iodine Checker, provide directions for the user to prepare the necessary reagents, which is less expensive than pre-measured reagent vials. [13] However, reagent preparation is technically demanding. It requires an analytical balance, volumetric pipettes, usage and cleaning of volumetric glassware and storage and handling of hazardous materials. If such facilities are available, the user could most likely conduct a standard iodometric titration instead.

### 4.4 Iodometric titration

Iodometric titration is the “gold standard” analytical method recommended for factory quality assurance/quality control (QA/QC) and research studies, but it is not practical for many small fortification facilities nor for field use in salt surveys. [5] The technique requires an analytical balance, burette, and preparation and standardization of reagents. The user must handle hazardous chemicals such as concentrated acids. These factors limit its use to a laboratory. Titration is one of the most accurate analytical methods available. An experienced technician using a well-developed titrimetric method can achieve 0.1% absolute accuracy with a relative standard deviation (RSD) of just 0.2%, showing high precision for the measurement. [14] Automatic titration instruments are commercially available, although not practical for field use in low-resource settings. In such settings, the accuracy and precision of titration are both significantly degraded. Use of over-concentrated titrants, impure reagents and uncalibrated or inaccurate balances or volumetric glassware contribute to this poor analytical performance. In a study conducted under field conditions in a developing country, the absolute accuracy of iodine titrations was off by an average of 14% and the measurement precision had an RSD of 10%. [13] The performance of titration compared with other field techniques is summarized in Table 1.

**Table 1.** Analytical metrics and costs of field-friendly, quantitative methods that analyse iodize salt. *Precision metrics were not reported in the study. ^#^Various iodine levels were tested multiple times and the precision for each level is reported.

Method	Accuracy	Imprecision	Cost (US$)
Rapid Test Kit[9]	72%	NA*	$0.01 per sample
Titration Under Field Conditions[13]	86%	10%	Lab setup $3,000 + $0.05 per sample
WYD Iodine Checker[13]	97%	6%	$400 initial investment + $1 per sample
BioAnalyt iCheck Iodine[12]	91%	1%	$3,500 initial investment + $3 per sample
ID-ERTK[4]	80%	8–13%^#^	cost not available

## 5. Other Methods

Potentiometry is used in well-established fortification facilities as well as analytical chemistry laboratories and the spectrophotometric measurement of the Sandell-Kolthoff (SK) reaction has been used by a few research institutes. [15] The SK reaction method requires a small sample portion (0.1 g) or numerous dilutions of a more representative sample portion (10 g) and generates arsenic-containing waste. [16] Both methods can be semi- or fully automated for processing large numbers of samples. However, these methods require sophisticated and expensive analytical instruments, well-equipped temperature-controlled laboratories, qualified scientists and technical staff to maintain and verify the equipment and operation of complex software programs. Thus they are unsuitable for field analysis and will not be further discussed.

### 5.1 SaltPAD

**Figure 1.**
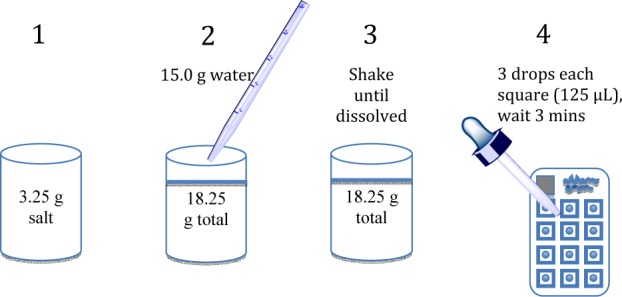
Instructions for how to use the test card to analyse a salt sample. Solid salt is mixed with water in the proportion of 3.25 g salt to 15.00 g water. This proportion provides a quantitative 1:5 dilution of the ppm iodine concentration in the solid after accounting for the density of the solution. A US$10 centigram balance provides sufficient accuracy for sample preparation. An automatic pipette can be used to apply 125 μL of solution to each square of the saltPAD. If an automatic pipette is not available, a 1 mL disposable plastic pipette, held perpendicular to the card surface, is used to place three drops of test solution onto each square of the saltPAD. If the solution does not immediately and spontaneously wet all five of the areas in the square, the pipette tip is drawn across the thin wax lines to allow wetting to occur. When all five areas are wetted by the solution, the surface tension of the drop forms a dome that provides a pathway for the stored reagents to dissolve, meet, and perform a threshold titration.

**Figure 2.**
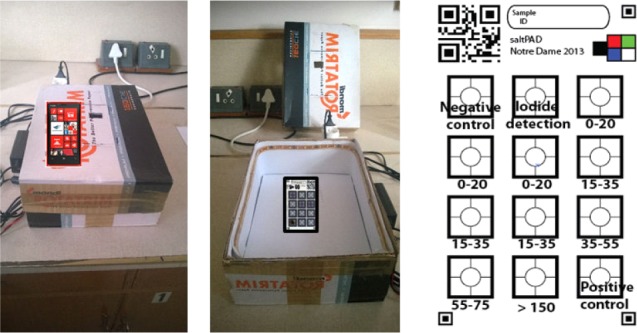
Illustration of the SAMRC light box and the positioning of the saltPAD in the box. The light box contains an LED strip light covered with a layer of white paper to diffuse the light. A Nokia Lumia 920 phone was used to capture the image of the saltPAD. The function and analytical range of each reaction area on the saltPAD is shown in the schematic on the right; each saltPAD is printed with a unique QR code, colour standards, and fiducial marks to facilitate image analysis.

A paper analytical device (the saltPAD) for analysis of iodized salt was recently developed with support from the Global Alliance for Improved Nutrition (GAIN) as a field-friendly alternative to glassware titration. [17] The saltPAD is a paper card printed with hydrophobic wax barriers that define 12 reaction areas. All of the reagents needed to perform a single point of an iodometric titration are stored in the reaction area. The user creates a test solution by mixing the salt sample with water, applies three drops of the solution to each of 12 reaction areas on the test card and then takes a photograph of the test card (Figure 1 and Figure 2). Each reaction area provides a small range in which iodine levels can be quantified due to the various levels of blue colour that are formed. By overlapping these quantitative ranges, the card can cover the analytically relevant range of concentrations found in iodized salt. An automated image analysis program interprets the colour response by comparing the card to a set of test cards run with standardized solutions. Alternatively, the user can compare the test card visually to stored images of test cards run with standard samples.

**Figure 3.**
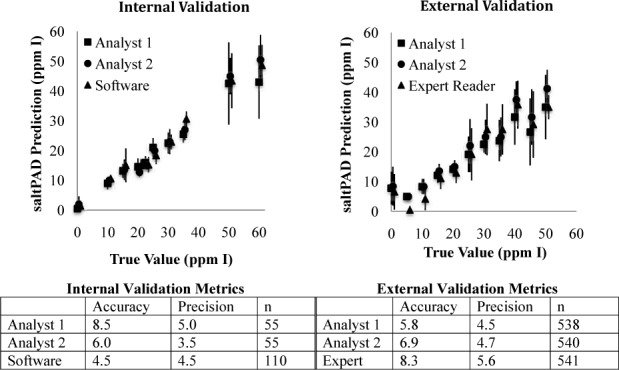
Internal and external validation of saltPAD. Accuracy and precision are expressed in units of mg of I atoms per kg iodized salt. The true values on the graphs have been slightly offset to ease the reading of the data. Since there are so many data points for the external validation, and to help ease the reading of the graph, only the data points with a true value that is a multiple of 5 ppm I have been displayed. The metric values in the table include all data points.

A blinded internal validation of the test card performance was carried out using solutions of 20% sodium chloride (NaCl) spiked with 11 levels of potassium iodate. These levels corresponded to the analytically relevant range of ppm levels of iodine in iodized salt. The internal validation established the accuracy as 7.2 ppm I (measured as mg I atoms/kg salt) and the precision as 4.5 ppm I when the saltPAD was prepared and read by newly trained users. Using computer software to process images, the accuracy improved to 4.5 ppm I and the precision remained the same. (Figure 3)

The first step towards implementation of a novel analytical device like the saltPAD is to determine whether it can be used outside the lab in which it was developed. GAIN set up and funded an external validation study, which was conducted by the South African Medical Research Council (SAMRC). A total of 287 salt samples were tested by titration and with the saltPADs. The data were analysed to determine the accuracy and precision of the saltPAD compared with titration, as well as the utility of the saltPAD for household or market surveys and for QC applications in salt fortification facilities.

The test cards were made at the University of Notre Dame (UND), vacuum-sealed in plastic and shipped to the SAMRC. The typical transit time was less than a week and the cards were used at between one and three months of age. The true values of the salt samples were blinded to the analysts running the saltPADs. For 108 samples, portions of non-iodized and iodized salts were mixed together in various ratios to get final iodine concentrations in the range of 5–50 ppm I. The remaining 179 samples were collected directly from the marketplace and contained 0–120 ppm I. The salt samples included fine, medium and coarsegrained salts. Two 10 g portions of each sample were iodometrically titrated and the average concentration was used as the true value for the study. The average RSD for titration was 5%, which reflects a combination of the sample heterogeneity and the titrimetric precision. Each salt sample was prepared for PAD analysis using 6.5 g of salt and 30 mL of deionized water and tested on two saltPADs. A picture of each saltPAD was taken in a light box (Figure 2). Two novice users at the SAMRC and an expert reader at UND analysed all of the images by visual comparison with standard images.

The average absolute accuracy was determined for each analyst (Figure 3); the average accuracy for all three analysts was 7.0 ppm I. The method precision for each analyst was calculated by taking the standard deviations of the duplicate analyses and averaging them; the average precision for all three analysts was 4.9 ppm I. These analytical metrics are basically the same as the metrics obtained in the internal validation study, showing that the saltPAD could function well in another laboratory setting.

**Figure 4.**
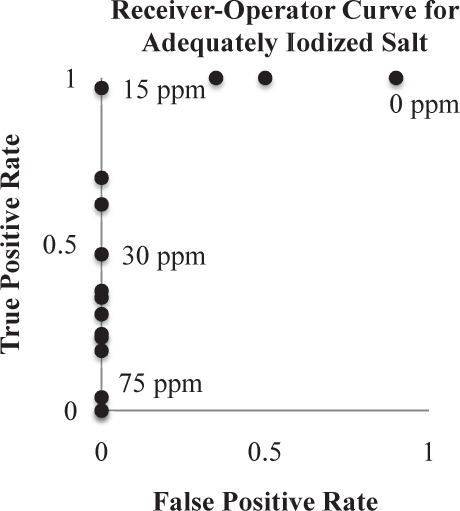
Receiver-operator curve for salt adequately iodized to 15 ppm. The area under the curve is 99.4%. The test card is extremely good at discriminating properly iodized salt from under-dosed salt.

The saltPAD design used in the SAMRC study was optimized to analyse whether or not samples in the marketplace contain at least 15 ppm of iodine. It performs triplicate measurements in the 0–35 ppm I range; see Figure 2. The card was designed so monitoring agencies could use it to perform surveys and the performance target is for >90% of the samples to be correctly categorized as iodine deficient (<15 ppm I) or iodine sufficient (≥15 ppm I). A cutoff point was set based on the results from the internal validation study and applied to the SAMRC saltPAD data. Figure 4 shows the receiver operating curve (ROC) plot for distinguishing adequately iodized salt (≥15 ppm) from inadequately iodized salt (<15 ppm). Three analysts each read 568 test cards (n = 1,704). Of the 486 under-iodized samples, 90.5% were correctly identified (Table 2). The saltPAD's performance for salt that was slightly under-iodized was still quite good; for the 174 samples in the 10–15 ppm iodine range, 86% were correctly identified as under-iodized. For the 1,218 samples with sufficient iodine levels, 92.1% were correctly identified as containing at least 15 ppm iodine.

**Table 2.** Accuracy table of the saltPAD as a market survey tool. More than 90% of under-iodized salt samples were correctly classified and more than 92% of adequately iodized salt samples were correctly classified.

Titration	saltPAD	
(ppm I)	<15	≥15
<10	290	22
10<x<15	150	24
≥15	96	1,122

Another possible use for a field-screening tool like the saltPAD is for workers to monitor the iodization process during salt fortification. The goal is to adjust the iodization machinery in close to real time. Although the saltPAD tested in the SAMRC study is optimized for detection of market-level iodization (with an analytical “sweet spot” around 15 ppm I), we evaluated whether this card would be suitable for quality control (QC) applications in a low-resource setting such as Kenya, where a usability study was under way (Table 3). Regulations in Kenya dictate that an iodized salt sample must be between 30–50 ppm I at the time of production in order to be sold in Kenyan marketplaces, so the process control specifications require detection of both a minimum and maximum level of iodine. For the 1,704 card images in the MRC study, only 76.2% of the visual interpretations produced correct categorization of the salt samples according to these QC levels. The high level of error is due to a systematic bias to low readings. However, only 1.4% of samples containing < 30 ppm I were predicted to have sufficient levels for sale, so it is unlikely that any salt iodized at levels that provide no health benefit (< 15 ppm) would make it to the market. The performance of this saltPAD would be improved by re-design to provide replicate measurements in the 30–50 ppm iodide range.

**Table 3.** Accuracy table of the saltPAD as a quality control tool. 76.2% of salt samples produced the correct categorization

Titration		saltPAD	
(ppm I)	<30	30–50	>50
<30	996	24	0
30–50	292	209	15
>50	13	62	93

To determine user-friendliness, a small study was performed at a salt fortification plant in Mombasa, Kenya. The plant uses spray deposition of potassium iodate solution, followed by mechanical drying and packaging. Salt travels on a conveyer belt underneath a spray nozzle. However, the spray rate must be adjusted based on many factors, including the temperature, the particle size of the salt and the conveyer belt speed. A technician grabs samples directly after the spray nozzle and performs titrations every 30–60 minutes. If the iodization level measured by titration is below 32 ppm I, the technician increases the spray rate and if the iodization level is above 46 ppm I, the technician decreases the spray rate.

One of the quality control technicians was trained to use the saltPAD, which took 31 minutes. Fourteen grab samples were collected over a period of several days. These samples were analysed with saltPADs in the salt fortification plant's titration lab. The laboratory temperature at the time of the study was 25–30°C with high humidity. The technician and two expert analysts visually interpreted the test card responses by comparison with standard images. The samples were also analysed by titration, but the results were kept blinded until after the saltPAD interpretation had been completed. The quantitative results from the novice and expert readers were statistically indistinguishable and the average of the differences between the saltPAD results and the titration results was 2 ± 6.5 ppm, which is statistically indistinguishable from zero (paired t-test, 95% CL). Performing the 28 saltPAD analyses took the technician three hours, which was the same time required to perform the 14 titrations (this does not include the time required to prepare and standardize the titration reagents). The saltPAD analyses generated less than 500 mL of waste (salt solutions plus the used saltPAD cards), while the titrations created over 5L of waste.

We evaluated the process changes that the saltPAD readings would have required the technician to perform, comparing them with the process changes required by the results of the gold standard titration analysis. The iodine level determined by titration in two of the salt samples was above 46 ppm and would require turning down the spray rate. Only 33% of the corresponding saltPAD readings indicated that the iodine level was too high. The iodine level for five of the salt samples was in the correct range and would not require any change in spray rate; 83% of the corresponding saltPAD readings indicated that the iodine level was in the correct range. The iodine level for seven of the salt sample cases was below 32 ppm and would require turning up the spray rate; the corresponding saltPAD readings indicated that the iodine level in 86% of these samples was too low. Overall, 77% of the saltPAD-based process actions matched those required by the titration results. The outcome from this study in a fortification facility was similar to the outcome from the SAMRC study, which was performed in a controlled laboratory environment.

A study in Burkina Faso was recently carried out to compare the performance of five quantitative field analysis technologies in a developing world setting (Tables 4 and 5).[4] Replicate analysis of spiked saline solutions and 59 salt samples from around the world was conducted. For each technology, analytical metrics such as accuracy and inter-device and inter-operator precision were determined. In addition, the usability and field-friendliness of each system was evaluated. Technologies were rated lower if they were difficult to perform correctly, required many steps or access to other lab equipment such as an analytical balance, if they generated hazardous waste, or if the technology required a controlled laboratory environment. All of the technologies except the saltPAD are commercially available systems based on the spectrophotometric analysis method. The commercial systems require that the user obtain and maintain the spectrometer as well as reagent solutions. For some of the spectrophotometric systems, the reagents must be prepared by the user and in other cases they are available as pre-measured portions for analysis of a single sample. The saltPAD was tested in a laboratory setting by experienced lab technicians using automatic pipettes for sample delivery, as well as in a field setting by non-technicians using disposable plastic droppers for sample delivery. The saltPAD was less accurate than some of the spectrophotometric systems, but was rated highly for being field- and user-friendly.

**Table 4.** [4] A comparative study by GroundWork ranked five technologies against each other for overall performance. All of the technologies except the saltPAD are commercially available.

Method Rank	Strengths	Weaknesses
I-Reader 1	Analytical metrics, user-friendly, field-friendly, compatible with low resource settings	Does not recommend appropriate sample preparation for heterogeneous samples
iCheck 2	Analytical metrics, user-friendly, field-friendly	Cost, needs computer, glass and sharps waste
saltPAD 3 (tied)	Field-friendly, user-friendly	Needs test kit development and automated image analysis
WYD 3 (tied)	Analytical metrics, compatible with low resource settings	Needs lab setting and skills
ID-ERTK 5	Compatible with low resource settings	Needs lab setting and skills

**Table 5.** Analytical metrics for the test card in various validation studies. All units are expressed as ppm iodine atoms in solid salt. *Inter-device/operator precision. **Study conducted by the South African Medical Research Council.

SUMMARY OF STUDIES			
Validation Type	# Samples	Accuracy (ppm I)	Precision* (ppm I)
Internal Lab[17]	107	4.5	4.7/2.4
External Lab**	1,619	7.9	1.4/2.3
Comparative[4]	203	7.1	1.6/6.2

**Figure 5.**
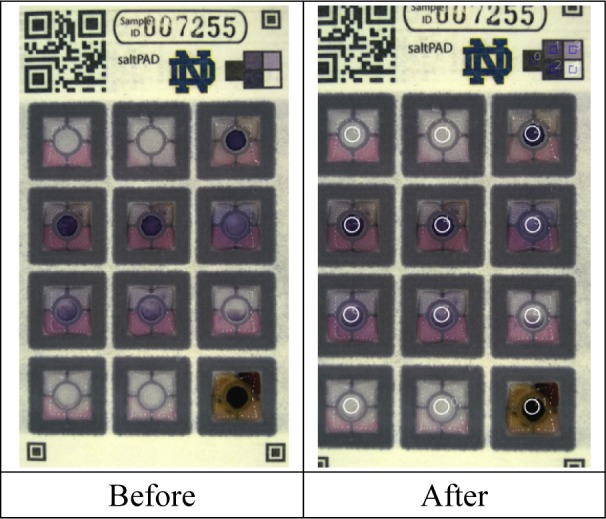
Automated software analysis images of the saltPAD. Before) A picture of a saltPAD taken with a cell phone. After) The input image is analysed by the program, which locates the regions of interest designated by the white circles. These coincide with the indicator spots. The greyscale intensity is measured, fitted to calibration curves stored in the program memory, and the resulting iodine concentration is reported to the user.

The saltPAD was the only device in the GroundWork study whose accuracy and precision improved when it was used by non-technicians: this was because two of the technicians in the central lab did not read the saltPAD results correctly. In order to eliminate this source of inter-operator variability, software was written to calculate a sample's iodine concentration from a cell phone image of the test card. A repository with the open source code for this image comparator is available at https://github.com/PaperAnalyticalDeviceND/SaltPad. This computer image analysis method makes use of fiducial marks and colour standards printed on the saltPAD (see Figure 2). First, the locations of six fiducial marks are determined and a geometrical correction is applied to remedy image scaling, tilt and keystoning. The image white balance is adjusted. A mask alignment step, shown in Figure 5, is used to identify the 12 reaction zones with their central indicator circles; these circles where colour development occurs are the regions of interest (ROIs) for the quantitative analysis of iodine concentration. The greyscale intensity integrated over each ROI is measured and then fitted to the appropriate calibration curve for that reaction zone, stored in the program memory. The program ignores any spots with colour intensities that fall outside the linear calibration ranges and rejects the card altogether if the positive or negative control spots give incorrect readings. The measured concentration and ROI data are output to the user in spreadsheet format. When this program was applied to the image data from the Burkina Faso study, it gave correct readings of the cards [4], eliminating the inter-operator imprecision.

## 6. Conclusions

The analytical metrics (accuracy and precision) of the saltPAD provide enough power to serve several important applications in low-resource settings, where the portability and low cost of the saltPAD could provide an advantage over other analytical methods. In particular, the saltPAD is well suited for quantitative household and market level surveys, where salt iodized at ≥15 ppm must be distinguished from salt iodized at lower levels. The saltPAD was able to correctly categorize more than 90% of the samples in the SAMRC study according to this criterion. Although titration is the “gold standard” for analysis of iodized salt, transporting household or market salt samples to a laboratory can introduce logistical delays of weeks or months and salt samples can also be lost or damaged, particularly if they are poorly packaged. An on-the-spot quantitative assay would eliminate these sources of delay and error. It would also provide an opportunity to discuss the results immediately with the market vendor or householder, which could be useful in supply chain surveillance or public health education contexts.

The saltPAD offers two features that are suited for low-resource settings. The mobile phone-based image analysis program provides a mechanism for collecting the test data centrally and allows a wide range of users to report accurate test results. Regulatory agencies and health organizations could use this feature to monitor iodized salt quality across a region in real time. Archiving, collating and comparing data could help to improve the transparency of monitoring efforts, as well as guard against bad practices such as dry labbing. Second, the capital cost for setting up saltPAD testing is very low, about US$20, and there is no requirement for the user to prepare reagents or provide any consumables other than the cards themselves. Although the cards are not yet available commercially, the cost is projected to be under US$1 per card. [17] This is in contrast with the US$3,000 cost of establishing a titration lab, which requires suitable laboratory space, an analytical balance, lab equipment and chemicals. It is also in contrast with the US$400-$4,000 capital outlay for a spectrophotometric reader with continuing costs of reagent preparation in a lab setting or purchase of US$1-4 per sample reagent vials.

Rapid test kits (RTKs) are inexpensive and simple to use, but do not meet the analytical needs for quantitative salt analysis. At best the existing rapid test kits used in surveys/MICS can only be used to qualitatively estimate broad categories of iodine concentrations. [9, 10] In contrast, the saltPAD could clearly distinguish between non-adequately iodized (< 15 ppm I) and adequately iodized salt (≥15 ppm I). Operation of the saltPAD is safer compared with the iCheck, which uses syringes with needles to transfer salt solutions and reagents from vial to vial. The saltPAD is a friendly, quantitative RTK that allows for rapid and simple analysis and is ideal for settings where sophisticated equipment is not available. This assay has been shown to produce reliable results in an internal study, an external laboratory and a fortification facility environment. However, it has not been tested under field conditions particular to health-related surveys and regulatory monitoring at border posts. Nevertheless, the current design of the saltPAD could replace qualitative rapid test kits used for many market and household surveys, providing better data on whether there is sufficient iodine present in salt samples.

The saltPAD design allows for rapid modifications to meet the specifications of various analytical tasks. The saltPAD's analytical “sweet spot”, which is the concentration range of greatest accuracy and precision, is determined by the number of replicate reaction areas provided in various iodine concentration ranges. For surveys whose only question is whether the salt sample is adequately iodized or not, the saltPAD could be redesigned with four sets of triplicate reaction areas that cover the 10–20 ppm range; hence four samples could be analysed on a single card, further reducing the cost per analysis. Alternatively, to accommodate fortification facilities and regulatory authorities, a saltPAD with higher sensitivity in the 30–50 ppm range (or other ranges as desired) could be designed, validated and manufactured. These options for modification of the sensitivity of the saltPAD could allow fortification facilities, without an established titration laboratory onsite, to monitor and improve the quality of iodized salt products.

Regardless of the card design, it would be desirable to fully automate the saltPAD reader as a software application for one of the many inexpensive smartphones now becoming available throughout the developing world. The smartphone would read the card's unique QR code, look up the appropriate calibration data and provide an immediate reading of the iodine concentration in the sample. It would also communicate the result to a central database, add timestamp and location data and prompt for direct entry of sample metadata. The modification of the saltPAD reader into a smartphone app is currently under way for a different paper analytical device in which iodometric titration is used to quantify beta-lactam antibiotics.

## 7. Compliance with Ethical Research Standards

NM and ML have applied for US patent 14533746, covering the paper test card described in this mss.

NM, ML, MAD, and EES received funding from the Global Alliance for Improved Nutrition (GAIN).

CS, JS, and RS declare no conflicts of interest.

No part of this study was performed on any human or animal subjects.
